# Vector dynamics influence spatially imperfect genetic interventions against disease

**DOI:** 10.1093/emph/eoaa035

**Published:** 2020-12-27

**Authors:** Mete K Yuksel, Christopher H Remien, Bandita Karki, James J Bull, Stephen M Krone

**Affiliations:** Department of Mathematics, University of Idaho, Moscow, ID 83844-1103, USA; Department of Mathematics, University of Idaho, Moscow, ID 83844-1103, USA; Department of Mathematics, University of Idaho, Moscow, ID 83844-1103, USA; Department of Biological Sciences, University of Idaho, Moscow, ID 83844-1103, USA; Department of Mathematics, University of Idaho, Moscow, ID 83844-1103, USA

**Keywords:** genetic pest management, gene drive, pathogen suppression, mosquito biting dynamics, spatial structure, mathematical model

## Abstract

**Background and objectives:**

Genetic engineering and similar technologies offer promising new approaches to controlling human diseases by blocking transmission from vectors. However, in spatially structured populations, imperfect coverage of the vector will leave pockets in which the parasite may persist. Movement by humans may disrupt this local persistence and facilitate eradication when these pockets are small, spreading parasite reproduction outside unprotected areas and into areas that block its reproduction. Here, we consider the sensitivity of this process to biological details: do simple generalities emerge that may facilitate interventions?

**Methodology:**

We develop formal mathematical models of this process similar to standard Ross–Macdonald models, but (i) specifying spatial structure of two patches, with vector transmission blocked in one patch but not in the other, (ii) allowing temporary human movement (travel instead of migration) and (iii) considering two different modes of mosquito biting.

**Results:**

We find that there is no invariant effect of disrupting spatial structure with travel. For both biting models, travel out of the unprotected patch has different consequences than travel by visitors into the patch, but the effects are reversed between the two biting models.

**Conclusions and implications:**

Overall, the effect of human travel on the maintenance of vector-borne diseases in structured habitats must be considered in light of the actual biology of mosquito abundances, biting dynamics and human movement patterns.

**Lay summary:** Genetic interventions against pathogens transmitted by insect vectors are promising methods of controlling infectious diseases. These interventions may be imperfect, leaving pockets where the parasite persists. How will human movement between protected and unprotected areas affect persistence? Mathematical models developed here show that the answer is ecology-dependent, depending on vector biting behavior.

## INTRODUCTION

Radically new technologies are becoming available to suppress vectored diseases. They operate as genetic modifications of vector populations that block parasite transmission. One such technology uses ‘modification’ gene drives that automatically sweep through the population. The drive is engineered to include one or more genes that interfere with the parasite in the vector [1–3]. A somewhat parallel approach, but without genetic engineering, introduces pathogen-blocking strains of the self-spreading bacterial symbiont *Wolbachia* into the vector [4, 5]. A third, and more mundane approach is to release huge numbers of lab-reared, genetically modified vectors, simply to infuse wild populations with transmission-blocking genes in a manner akin to the sterile insect technique [6, 7]. The gene drive and *Wolbachia* approaches result in possibly permanent alterations of vector populations because the genetic modifications are selectively maintained. The swamping method is typically transient, because the modification is not coupled with any selective benefit [7]; continual releases of engineered vectors would be required to maintain the parasite block.

Genetic modifications have an advantage in that they accrue directly and specifically to the vector and are transmitted intact to offspring, contrasting with pesticides that are broadcast environmentally, cannot be uniformly applied and need to be applied repeatedly. However, genetic methods are sometimes controversial and face extreme regulatory hurdles because of their transgenerational permanence. We nonetheless imagine that many of these genetic technologies will be widely implemented in the near future; indeed, *Wolbachia* and genetically engineered sterile males are already in use. Thus, predicting the possible bases of failure versus success may be useful in ensuring the best possible outcomes. Some methods may seem so foolproof as to ensure disease eradication because of their ability to modify huge fractions of vector populations. Even so, one worry is that any population intervention is likely to be incomplete, leaving spatial pockets of minimal coverage interspersed with perhaps large pockets of almost total coverage [8, 9]. What will be the effect of these pockets of poor coverage?

If parasites are suppressed almost everywhere throughout their range by a genetic intervention, the disease burden will decline markedly and perhaps for the long term. But parasites persisting even in small regions, even though they may have a small impact on overall disease burden, become a source for future parasite recovery and may also foster the evolution of resistance to the intervention. These reservoirs may thus require secondary interventions for eradication, so understanding what factors enable parasite persistence in these reservoirs may prove crucial in applying those secondary interventions.

From a greatly simplified spatial model of pathogen dynamics, we previously suggested that spatial structure will foster the persistence of the pathogen in small pockets when the pathogen would disappear in the absence of structure [10]. Thus, any softening of spatial structure would help limit parasite persistence—suggesting a possible intervention in the form of host movement. That model omitted vectors as well as hosts, so any inference to vector dynamics was tangential. Here, we consider a more biological model of spatial structure than we addressed previously: a model that includes vectors, with host mobility. When hosts are spatially clustered and a genetic intervention blocks vector transmission most places, does host movement invariably facilitate eradication? Furthermore, how does the effect of human movement depend on the transmission dynamics?

Aspects of our problem have been addressed in prior mathematical studies of vectored diseases. The original and most prominent models of vectored disease are the Ross–Macdonald models [11]. The effect of spatial structure on disease dynamics has been addressed in several modeling studies when assuming a single model of transmission dynamics [12–17]. The effect of different models of transmission dynamics has been addressed in the absence of spatial structure [18, 19]. Our models combine spatial structure, differential blocking of transmission among patches, human movement among patches and different forms of mosquito biting dynamics. Our assemblage of assumptions is unique, but this broad foundation of previous work simplifies our task and provides many anchor points to validate our findings.

## RESULTS

### Foundations

The Introduction provided several biological contexts for the problem we study. They all involve vectored infectious diseases, spatial structure and movement of vectors and/or humans (we consider only the latter here). Here, we explain how that biology is converted into our models.

### Population structure

Our models are standard epidemiological ‘SIS’ models, accounting for vector (mosquito) and host (human) numbers, as well as spatial structure. Parasites have no individual existence *per se* in the model; they exist only as infected states of mosquitoes or humans. Infections are transmitted only mosquito to human or human to mosquito. A full description of the mathematical models is given in the Appendix.

To abstract this biological process, we model a population with discrete subpopulations; the same population subdivisions coincide for both humans and vectors, but it operates somewhat differently for humans than for vectors. The number of human residents in each patch is invariant; no one is born and no one dies during the time period considered. In contrast, mosquitoes have a patch-specific birth rate (independent of the number of mosquitoes and humans) and a patch-invariant death rate, leading to a patch-specific equilibrium density; mosquito lifetimes, on the order of weeks or months, are much shorter than human lifetimes. Mosquito spatial structure is rigid and invariant, whereas humans have a home patch but travel temporarily to non-resident locations—a movement scheme that differs from formal ‘migration’ [12]. The state of mosquito infections at a location depends on mosquito behavior and on the history of their exposure to humans at that location, regardless of whether the humans were residents or visitors. In contrast, humans are not confined to one location throughout life; they move, but each person is identified with a home residence, regardless of their location at any moment. This process would arise with daily commuting, jobs that involve travel, and even some kinds of nomadic lifestyles. (Our approach thus differs from standard migration models in which individuals move without memory of an individual’s previous residence.) Because humans travel, their infection status depends on their history of exposure to mosquitoes at the different locations they have occupied.

### Transmission dynamics

**Figure 1.**
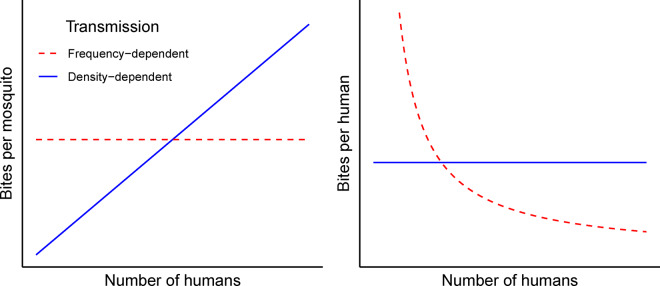
Differences between the FD and DD models with respect to biting dynamics. The left panel shows biting rate per mosquito, the right panel shows biting rate per human. The solid (blue) lines apply to the DD case, dashed (red) to the FD case.

We consider two models of infection dynamics as they affect mosquito biting rates: density-dependent (DD) and frequency-dependent (FD) [18, 19]. These models differ in the way the biting rate of mosquitoes at a site scales with the number of humans at that site (Fig. 1). In the DD model, characterized by a mass-action functional response, the rate at which a single person is bitten is independent of the number of humans; in the FD model, characterized by a saturated functional response, the total number of bites is determined by the number of mosquitoes, so adding more humans decreases the bite rate per person unless mosquito density increases with human density. The standard Ross–Macdonald models often assume frequency-dependence.

Our main result will be that human densities and visitation parameters have fundamentally different effects in the DD and FD biting models. To facilitate understanding that conclusion, we explain up front the key model differences that account for those results. The differential equations that describe transmission dynamics for the two models (see Appendix) each have a constant biting rate. The rate is simply $b_{\text{DD}}$ per mosquito per human in the DD model. But in the FD model, this biting rate is of the form $b_{\text{FD}}/{\tilde{H}}^{\left(j\right)}$ in patch *j*, where ${\tilde{H}}^{\left(j\right)}$ represents the effective number of humans in patch *j* based on residents who stay in the patch and visitors from the other patch. In the contrived case with patch sizes and visitation parameters lining up to produce ${\tilde{H}}^{\left(1\right)}={\tilde{H}}^{\left(2\right)}$ , we could set $b_{\text{DD}}=b_{\text{FD}}/{\tilde{H}}^{\left(1\right)}$ to get identical dynamics between the two models. As we vary patch size and visitation parameters across different simulations, however, major differences between the DD and FD models become apparent. In the DD case, if mosquito density is the same across patches, each human is bitten at the same rate regardless of patch. Whereas in the FD case, humans are bitten at a lower rate in the patch with more humans (again for constant mosquito densities).

### ${ℛ}_{0}$ calculations when transmission is blocked in one patch.

With vectored diseases, there are various ways to calculate the basic reproduction number, ${ℛ}_{0}$ [11, 15]. Our method (which can be found in the Appendix) is essentially that of [11]. For our purposes, the actual value of ${ℛ}_{0}$ is unimportant, as we are interested in the relative impact on ${ℛ}_{0}$ of changes in population structure, as well as a relative comparison of ${ℛ}_{0}$ for density dependence and frequency dependence. Typically, different methods of computing basic reproduction numbers in vector models can lead to different ${ℛ}_{0}$ values (e.g. one value being the square of what is obtained via a different method) but they agree at the epidemic threshold of ${ℛ}_{0}=1$ , which again is the critical value between eradication and endemism.

To keep the focus on biological relevance, we limit consideration to two patches. As per our biological justification above, we let the intervention be fully effective and block all transmission in patch 1, but the intervention is absent in patch 2. Maintenance of the parasite (${ℛ}_{0}>1$ ) in this setting is due entirely to whether the parasite persists in patch 2.

We wish to consider conditions whereby, in the absence of human movement, the parasite would persist in patch 2. The question is then whether and how movement affects persistence. Our previous analysis which neglected hosts and vectors, [10] can be construed to suggest that, if patch 1 was sufficiently large, human movement between patches would facilitate parasite eradication by increasingly exposing the parasite to the average of both patches (as also true of [13]). We are interested in whether this conclusion holds in models that include both humans and vectors: how does human movement affect persistence and how do the two models compare?

The ${ℛ}_{0}$ formula for either model (DD or FD) is a function of seven parameters and three state variables (derived for general transmission values in the Appendix). For the FD model with no mosquito-to-human transmission in patch 1, the formula is

(1) $${ℛ}_{0}^{\text{FD}}=\frac{\left[b_{\text{FD}}^{2}\cdot a_{\text{MH}}^{\left(2\right)}\cdot a_{\text{HM}}\cdot M^{\left(2\right)}\right]\cdot \left[c_{22}^{2}\cdot H^{\left(2\right)}+c_{12}^{2}\cdot H^{\left(1\right)}\right]}{\gamma \cdot \delta \cdot {\left[c_{22}\cdot H^{\left(2\right)}+c_{12}\cdot H^{\left(1\right)}\right]}^{2}}\;(\text{frequency}\;\text{dependent}),$$

with notation defined in Table 1. The first numerator term in brackets is a mosquito term that accounts for the number of mosquitoes, transmission rates per bite in both directions, and biting rates; the squared biting rate accounts for the mosquito acquisition of the parasite and then its later transmission. The second numerator term in brackets is one of human population size weighted by (squared) human travel probabilities to account for only those humans present in patch 2—the patch with no block to transmission. (Note that the constraints $c_{21}+c_{22}=c_{11}+c_{12}=1$ mean that the formula in (1) specifies all movement patterns.) The denominator is a squared term of humans present in patch 2, necessarily larger than the human term in the numerator given moderate to high human densities (given that the $c_{ij}\leq 1$). Inspection of this result reveals how increasing the numbers of humans in patch 2, while holding the mosquito term constant, reduces ${ℛ}_{0}^{\text{FD}}$, reflecting the dilution of mosquito bites. These results have been confirmed with limited numerical analyses of the full equations by varying *c*_12_ and *c*_21_. The threshold ${ℛ}_{0}=1$ in (1) (and (2) below) coincided with the threshold for maintenance or loss of the parasite.

**Table 1.** Description of state variables and parameters in the mathematical models, which are described in the Appendix.

Notation	Description	Units
$H^{\left(k\right)}$	Number of human residents in patch *k*	individuals
$M^{\left(k\right)}$	Number of mosquitoes in patch *k*	individuals
*γ*	Recovery rate of infected humans	day^−1^
*b*_DD_	Density-dependent biting rate	individual^−1 ^ day^−1^
*b*_FD_	Frequency-dependent biting rate	day^−1^
*δ*	Mosquito death rate	day^−1^
*c_kj_*	Fraction of time patch *k* humans spend in patch *j*	dimensionless
*a*_HM_	Human-to-mosquito transmission probability	dimensionless
$a_{\text{MH}}^{\left(k\right)}$	Patch *k* mosquito-to-human transmission probability	dimensionless

What is of greater interest here is the comparison of ${ℛ}_{0}$ values between the DD and FD models. For the DD model,

(2) $${ℛ}_{0}^{\text{DD}}=\frac{\left[b_{\text{DD}}^{2}\cdot a_{\text{MH}}^{\left(2\right)}\cdot a_{\text{HM}}\cdot M^{\left(2\right)}\right]\cdot \left[c_{22}^{2}\cdot H^{\left(2\right)}+c_{12}^{2}\cdot H^{\left(1\right)}\right]}{\gamma \cdot \delta }(\text{density}\;\text{dependent}).$$

Note that the mosquito biting rate term here has different units than in (1)—see Table 1. Also note that there is no denominator term involving humans. In the following section, we compare ${ℛ}_{0}^{\text{DD}}$ and ${ℛ}_{0}^{\text{FD}}$.

### Travel has different effects under frequency dependence versus density dependence

There are obvious similarities in the DD and FD ${ℛ}_{0}$ formulae, and we may compare them as follows:

(3) $${ℛ}_{0}^{\text{FD}}={ℛ}_{0}^{\text{DD}}\cdot {\left(\frac{b_{\text{FD}}}{b_{\text{DD}}}\right)}^{2}\cdot {\left[\frac{1}{\left(c_{22}\cdot H^{\left(2\right)}+c_{12}\cdot H^{\left(1\right)}\right)}\right]}^{2}.$$

**Figure 2.**
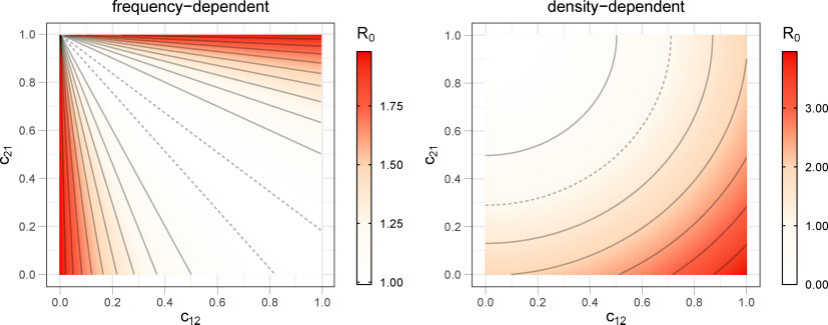
Contour plots of the basic reproduction number as a function of the visitation parameters (*c*_12_ and *c*_21_) reveal a fundamentally different effect of human movement under frequency dependence (left) than under density dependence (right). In each panel, the dashed contour line represents ${ℛ}_{0}(c_{12},c_{21})=1$, solid curves represent other values. These plots used a single set of parameters except for the *c_ij_*, but plots using other values are similar, except the curvature (and steepness in the FD case) of the contour lines change when other parameters are varied. Values of the parameters used are $\gamma =0.071,\;\lambda_{1}=50$  $\lambda_{2}=50,\;a_{\text{MH}}^{\left(1\right)}=0,\;a_{\text{MH}}^{\left(2\right)}=0.5$, *a*_HM_ = 0.8, δ=0.02, *b*_DD_ = 0.000075 and *b*_FD_ = 0.0375. The population sizes used are $H^{\left(1\right)}=H^{\left(2\right)}=500$ and $M^{\left(2\right)}=2500.$ Calculations were done using expressions (1) and (2) for the basic reproduction numbers. The constraints $c_{21}+c_{22}=c_{11}+c_{12}=1$ mean that the figures here specify all movement patterns between patches.

The difference of greatest biological interest is in the rightmost term of (3) when considered along with (1) and (2). With increasing numbers of humans in patch 2 (while maintaining constant mosquito density), the ${ℛ}_{0}$ for frequency dependence declines, whereas the ${ℛ}_{0}$ for density dependence increases. Note that increasing the number of humans in patch 2 can be accomplished by either increasing *c*_22_ or increasing *c*_12_. Increasing *c*_22_ increases spatial structure globally, whereas increasing *c*_12_ reduces spatial structure. The very different effects of human movement on ${ℛ}_{0}^{\text{FD}}$ and ${ℛ}_{0}^{\text{DD}}$ are illustrated in Fig. 2.

For the goal of parasite eradication, which in both models requires its eradication in patch 2, the contrast between the FD and DD models is extreme when considering spatial structure of humans. Reducing travel out of patch 2 increases ${ℛ}_{0}$ in the DD case but decrease ${ℛ}_{0}$ in the FD case. There are also contrasting effects of travel into patch 2 (*c*_12_). Thus not only do the FD and DD models differ in the effect of changes in effective patch size (number of humans), but the effect of changing spatial structure can differ between the models depending on whether travel involves humans leaving patch 2 or coming into it.

One of the most surprising, counterintuitive features of the model with FD transmission is the lack of dependence of the basic reproduction number on travel from the unprotected to protected patch (*c*_21_) when there is no movement in the other direction ($c_{12}=0$ ). This outcome is merely one of the extreme consequences of frequency dependence: mosquito biting rates fully adjust to human density to maintain the same transmission. Double bites and, thus, repeated infections in the unprotected patch are increasingly common in the FD regime as the human population decreases in size. In this instance, no matter the degree of movement to the patch where transmission is suppressed, the disease always persists among hosts to the same extent in the unprotected patch.

## DISCUSSION

Our study is motivated by new technologies that are being used or will likely be used as interventions against vectored infectious diseases. They involve genetically modifying the vector to block its competence for parasite reproduction or transmission. As it is unlikely that any such interventions will cover entire vector populations, our interest lies in the consequences of unprotected vectors. In the absence of spatial structure, the overwhelming abundance of modified vectors would suppress the parasite, but with strong spatial structure, unprotected pockets/patches of vectors will enable the parasite to persist. What, then, is the effect of limited disruption of that spatial structure, as in the form of human travel into and out of those pockets of persistence?

Our main result is simple: the effect of disruption to spatial structure (human travel) varies with changes in assumptions about mosquito biology. There is no general effect of human movement that transcends biological details. Outcomes depend on fundamental properties of model structure rather than stemming from nuances of parameter values.

Although our conclusion is easily stated, it is neither simple to demonstrate nor especially intuitive. We studied two types of well-established mathematical models of host-vector parasite dynamics. One model is a form of the long-used Ross–Macdonald model [11, 13, 14, 16, 18], a model that assumes FD behavior of mosquito biting. Frequency dependence is characterized by individual mosquitoes biting at a fixed rate, less per person as the local human population increases. Our other model is similar except in assuming DD biting rates; here individual humans are bitten at the same rate per mosquito regardless of how many people there are. All models assumed two patches of humans and their resident mosquitoes; the mosquitoes in one patch were blocked from transmission, but the mosquitoes in the other patch were fully competent. With strict spatial structure (no human movement), the parasite would be completely absent in one patch but present at high levels in the other patch.

The findings challenge our initial prejudices. From a casual consideration of previous work [10, 13], we expected that any relaxation of spatial structure would reduce the disease ${ℛ}_{0}$ if the disease-free patch was large enough relative to the diseased patch. Thus, sufficient human movement between the patches would eventually cause parasite extinction. We likewise did not anticipate fundamentally different behaviors of the FD and DD models: indeed, if population sizes are the same in each patch, the two models lead to identical dynamics (provided the relationship between $b_{\text{DD}}$ and $b_{\text{FD}}$ is chosen appropriately). It is only when varying human or mosquito densities in specific ways that the behaviors diverge.

The results here were therefore unexpected in that (i) different directions of movement (travel into or out of the patch) had opposing effects in a model, and (ii) those opposing effects were sometimes reversed between the two types of model. In hindsight, differences between the two models are understandable by considering the effect of increasing human density in a patch. In the DD model, an increase in humans in a patch results in more mosquito biting (per mosquito) and thus more disease transmission; in the FD model, mosquitoes do not increase biting activity so added humans results in a ‘swamping’ effect where most humans are protected due to the presence of other humans. These contrasting effects of the two models are at least broadly compatible with prior analyses that discovered opposing effects of movement on ${ℛ}_{0}$ between FD and DD assumptions in single-population models [19].

Transmission is the critical process, and it is driven by the mosquito biting rate, which may vary with human and mosquito densities in a variety of ways depending on the ecology specific to each population [18]. To simplify the analysis, we omitted human birth/death and fixed mosquito birth/death parameters within a patch, independently of human density. The difference between density and frequency dependence was then invoked in the mosquito biting rate per human in the patch. The models might have achieved similar effects by instead varying mosquito abundance in response to the number of humans present, while holding biting rates the same per mosquito. Indeed, a FD model that assumed mosquito density increasing with human density would effectively be invoking a type of density dependence in which mosquito biting rate per human keeps up with increases in human population size. There are thus many different formulations possible for mosquito biting per changes in human density, and an obvious next step in applying these models is to understand the biology so it can be better modeled.

A couple of alternative models of mosquito biting have in fact been analyzed [20, 21]. A single model of mosquito birth/death and biting dynamics can accommodate both density and FD biting rates as extremes, with high mosquito densities relative to humans tending toward density dependence, low densities tending toward frequency dependence [20]. Thus there is no *a priori* basis for one model being intrinsically more biologically plausible than the other, or indeed than either of these models being more plausible than some other more complex formulation of transmission dynamics. One advantage of analyzing two distinct models is that the fundamental effect of mosquito biting biology was easily illustrated by comparing the extremes.

The potential complexities of mosquito biting biologies do not end here: density and frequency dependence may differentially accrue to humans and vectors or may apply differently in different patches. It is unfortunate that the model outcomes are sensitive to seemingly minor changes biological assumptions—and presumably difficult properties to measure. Minimally, the models here caution against reliance on untested properties of mosquito dynamics and behavior; the biology is critical.

A broad context for our results is that the scale of spatial structure affects the persistence and abundance of parasites. This general class of result is known from other interventions. One example is of Bt-engineered crops that are used to kill the pests that would otherwise eat the crops [22, 23]. Even before Bt crops were introduced, it was known that resistance to Bt toxins circulated at low frequency in pest populations. The worry was that high levels of Bt-resistance would quickly evolve from this pre-existing resistance. However, it was discovered that the ascent of resistance could be greatly delayed by growing ‘refuges’ of Bt-free crops adjacent to Bt-expressing crops to overwhelm any Bt-resistant pests by matings with Bt-sensitive insects from the refuges. In this case, the relevant spatial structure is the distance that the pests move in seeking mates. In a second intervention, strains of the bacterial symbiont *Wolbachia* are introduced into mosquitoes to block dengue virus transmission [5, 24, 25]. The *Wolbachia* can ultimately spread throughout the mosquito population if introduced at a sufficiently high local density. In this case, spatial structure greatly facilitates attaining the threshold density locally, from which the *Wolbachia* can continue to spread. But spatial structure has also the undesirable effect of slowing symbiont spread.

Our models are not of evolution per se, but they are relevant to parasite evolution. First and most obviously, pockets of parasite persistence following a genetic intervention do not imply that the parasite has evolved resistance. Instead, persistence may be a consequence of spatial structure and appropriate forms of human or vector movement. Second, the populations of parasites that do persist may nonetheless become sites for evolution of resistance. This second point provides impetus for eliminating these pockets, which in turn motivates understanding how they persist.

Joint spatial structure of both vectors and humans is likely to present a major challenge to disease eradication by genetic modification of populations. Even with seemingly perfect blocking by the genetic engineering in those regions where coverage is high, potentially small unaltered vector populations will allow parasite maintenance provided the humans and vectors remain appropriately structured. The work here points to previously unappreciated complexities in the final steps of eradicating parasite refuges escaping these interventions. With this understanding, it may become possible to design secondary interventions that specifically target the pockets of escape.

## APPENDIX

### Two formulations of two-patch vector-human models with cross-patch visits by humans: DD versus FD transmission

Our models, written as systems of ordinary differential equations, track densities of susceptible and infected humans and mosquitoes in two patches connected by human movement. We let $H_{s}^{\left(k\right)}$ and $H_{i}^{\left(k\right)}$ be the densities of susceptible and infected human hosts in patch k∈{1,2} and $M_{s}^{\left(k\right)}$ and $M_{i}^{\left(k\right)}$ be the densities of susceptible and infected mosquitoes in patch *k*. The difference between the DD (density-dependent) and FD (frequency-dependent) models is encapsulated in the mosquito ‘biting rates’. For the DD model, $b_{\text{DD}}$ denotes the rate of biting per human per day by a given mosquito. In the FD model, $b_{\text{FD}}/H$ denotes the rate of biting per human per day by a given mosquito when the (local) density of humans is *H*. In other words, a given mosquito doles out $b_{\text{DD}}H$ bites per day in the DD model, and $b_{\text{FD}}$ bites per day in the FD model. (When the number of humans increases, DD mosquitoes work harder; FD mosquitoes do not change their biting rate, but must allocate their bites among more humans.)

The probability that an uninfected human becomes infected when bitten by an infected mosquito from patch *k* is given by $a_{\text{MH}}^{\left(k\right)}$. Dependence on the patch of the infecting mosquito reflects the assumptions that the level of parasite suppression is patch-dependent (as when the intervention is present in one patch but not the other) and an absence of mosquito movement among patches. Human-to-mosquito transmission is characterized by the parameter $a_{\text{HM}}$, which denotes the probability that an uninfected mosquito becomes infected when it bites an infected human from either patch; there is no patch-specific interference of human-to-mosquito transmission. Said differently, patch-specific heterogeneity in transmission probability (and thus transmission rate) of the disease from mosquitoes to humans is what characterizes the effectiveness of the genetic intervention. Owing to the focus of intervention efforts on the transmission from vector to human host, there is no such need to introduce patch-specific differences in human-to-mosquito transmission.

Let *δ* denote the death rate of mosquitoes and *γ* the recovery rate of an infected human. We also let *λ_k_* be the birth rate of susceptible mosquitoes in patch *k*. The equilibrium density of mosquitoes in patch *k* is, thus, given by $\lambda_{k}/\delta$. We focus on parasite transmission dynamics when mosquito density is constant. The fraction of time a human residing in patch 1 spends in patch 1 (resp., patch 2) is denoted by *c*_11_ (resp., *c*_12_), where $c_{11}+c_{12}=1$. Similarly, human residents of patch 2 spend fractions *c*_21_ and *c*_22_ in patches 1 and 2. Note that our human movement model is one of ‘visitation’ rather than actual migration. An example would be people who commute between their home city and another for work. We assume that $0\leq c_{12}<1$ and $0\leq c_{21}<1$ to ensure that there are actually people in each patch.

### DD transmission

In the case of DD transmission, infection rates for mosquitoes and humans have a mass-action dependence on mosquito and human densities.

$$\begin{array}{l} {\dot{H}}_{s}^{\left(1\right)}=-b_{\text{DD}}H_{s}^{\left(1\right)}\left[c_{11}a_{\text{MH}}^{\left(1\right)}M_{i}^{\left(1\right)}+c_{12}a_{\text{MH}}^{\left(2\right)}M_{i}^{\left(2\right)}\right]\;+\gamma H_{i}^{\left(1\right)}\\ {\dot{H}}_{i}^{\left(1\right)}=\;b_{\text{DD}}H_{s}^{\left(1\right)}\left[c_{11}a_{\text{MH}}^{\left(1\right)}M_{i}^{\left(1\right)}\;+c_{12}a_{\text{MH}}^{\left(2\right)}M_{i}^{\left(2\right)}\right]\;-\gamma H_{i}^{\left(1\right)}\\ {\dot{M}}_{s}^{\left(1\right)}=\;\lambda_{1}-b_{\text{DD}}a_{\text{HM}}M_{s}^{\left(1\right)}\left[c_{11}H_{i}^{\left(1\right)}+c_{21}H_{i}^{\left(2\right)}\right]\;-\delta M_{s}^{\left(1\right)}\\ {\dot{M}}_{i}^{\left(1\right)}=\;b_{\text{DD}}a_{\text{HM}}M_{s}^{\left(1\right)}\left[c_{11}H_{i}^{\left(1\right)}+c_{21}H_{i}^{\left(2\right)}\right]-\delta M_{i}^{\left(1\right)}\\ {\dot{H}}_{s}^{\left(2\right)}=\;-b_{\text{DD}}H_{s}^{\left(2\right)}\left[c_{21}a_{\text{MH}}^{\left(1\right)}M_{i}^{\left(1\right)}+c_{22}a_{\text{MH}}^{\left(2\right)}M_{i}^{\left(2\right)}\right]\;+\gamma H_{i}^{\left(2\right)}\\ {\dot{H}}_{i}^{\left(2\right)}=\;b_{\text{DD}}H_{s}^{\left(2\right)}\left[c_{21}a_{\text{MH}}^{\left(1\right)}M_{i}^{\left(1\right)}+c_{22}a_{\text{MH}}^{\left(2\right)}M_{i}^{\left(2\right)}\right]-\gamma H_{i}^{\left(2\right)}\\ {\dot{M}}_{s}^{\left(2\right)}=\;\lambda_{2}\;-b_{\text{DD}}a_{\text{HM}}M_{s}^{\left(2\right)}\left[c_{22}H_{i}^{\left(2\right)}+c_{12}H_{i}^{\left(1\right)}\right]\;-\delta M_{s}^{\left(2\right)}\\ {\dot{M}}_{i}^{\left(2\right)}=\;b_{\text{DD}}a_{\text{HM}}M_{s}^{\left(2\right)}\left[c_{22}H_{i}^{\left(2\right)}+c_{12}H_{i}^{\left(1\right)}\right]\;-\delta M_{i}^{\left(2\right)}. \end{array}$$

### FD transmission

Let $H^{\left(k\right)}=H_{s}^{\left(k\right)}+H_{i}^{\left(k\right)}$ denote the total number of humans who reside in patch *k*. Then, for example, mosquitoes residing in patch 1 will see a mix of humans: $c_{11}H^{\left(1\right)}$ residents of patch 1 who are not visiting patch 2, and $c_{21}H^{\left(2\right)}$ residents of patch 2 who are visiting patch 1. The ‘effective’ number of humans in patch 1 (i.e. the number of humans experienced by mosquitoes in patch 1) is thus ${\tilde{H}}^{\left(1\right)}\equiv c_{11}H^{\left(1\right)}+c_{21}H^{\left(2\right)}$. Similarly, the effective number of humans in patch 2 is ${\tilde{H}}^{\left(2\right)}\equiv c_{12}H^{\left(1\right)}+c_{22}H^{\left(2\right)}$. In the FD transmission framework, a mosquito’s bites are randomly allocated to this mix of humans.

$$\begin{array}{c} {\dot{H}}_{s}^{\left(1\right)}=-b_{\text{FD}}H_{s}^{\left(1\right)}\left[\frac{c_{11}a_{\text{MH}}^{\left(1\right)}M_{i}^{\left(1\right)}}{{\tilde{H}}^{\left(1\right)}}+\frac{c_{12}a_{\text{MH}}^{\left(2\right)}M_{i}^{\left(2\right)}}{{\tilde{H}}^{\left(2\right)}}\right]+\gamma H_{i}^{\left(1\right)}\\ {\dot{H}}_{i}^{\left(1\right)}=b_{\text{FD}}H_{s}^{\left(1\right)}\left[\frac{c_{11}a_{\text{MH}}^{\left(1\right)}M_{i}^{\left(1\right)}}{{\tilde{H}}^{\left(1\right)}}+\frac{c_{12}a_{\text{MH}}^{\left(2\right)}M_{i}^{\left(2\right)}}{{\tilde{H}}^{\left(2\right)}}\right]-\gamma H_{i}^{\left(1\right)}\\ {\dot{M}}_{s}^{\left(1\right)}=\lambda_{1}-b_{\text{FD}}a_{\text{HM}}M_{s}^{\left(1\right)}\cdot \left[\frac{c_{11}H_{i}^{\left(1\right)}+c_{21}H_{i}^{\left(2\right)}}{{\tilde{H}}^{\left(1\right)}}\right]-\delta M_{s}^{\left(1\right)}\\ {\dot{M}}_{i}^{\left(1\right)}=b_{\text{FD}}a_{\text{HM}}M_{s}^{\left(1\right)}\cdot \left[\frac{c_{11}H_{i}^{\left(1\right)}+c_{21}H_{i}^{\left(2\right)}}{{\tilde{H}}^{\left(1\right)}}\right]-\delta M_{i}^{\left(1\right)}\\ {\dot{H}}_{s}^{\left(2\right)}=-b_{\text{FD}}H_{s}^{\left(2\right)}\left[\frac{c_{22}a_{\text{MH}}^{\left(2\right)}M_{i}^{\left(2\right)}}{{\tilde{H}}^{\left(2\right)}}+\frac{c_{21}a_{\text{MH}}^{\left(1\right)}M_{i}^{\left(1\right)}}{{\tilde{H}}^{\left(1\right)}}\right]+\gamma H_{i}^{\left(2\right)}\\ {\dot{H}}_{i}^{\left(2\right)}=b_{\text{FD}}H_{s}^{\left(2\right)}\left[\frac{c_{22}a_{\text{MH}}^{\left(2\right)}M_{i}^{\left(2\right)}}{{\tilde{H}}^{\left(2\right)}}+\frac{c_{21}a_{\text{MH}}^{\left(1\right)}M_{i}^{\left(1\right)}}{{\tilde{H}}^{\left(1\right)}}\right]-\gamma H_{i}^{\left(2\right)}\\ {\dot{M}}_{s}^{\left(2\right)}=\lambda_{2}-b_{\text{FD}}a_{\text{HM}}M_{s}^{\left(2\right)}\left[\frac{c_{22}H_{i}^{\left(2\right)}+c_{12}H_{i}^{\left(1\right)}}{{\tilde{H}}^{\left(2\right)}}\right]-\delta M_{s}^{\left(2\right)}\\ {\dot{M}}_{i}^{\left(2\right)}=b_{\text{FD}}a_{\text{HM}}M_{s}^{\left(2\right)}\left[\frac{c_{22}H_{i}^{\left(2\right)}+c_{12}H_{i}^{\left(1\right)}}{{\tilde{H}}^{\left(2\right)}}\right]-\delta M_{i}^{\left(2\right)}. \end{array}$$

### DD ${ℛ}_{0}$ calculations

The basic reproduction number, especially for vectored disease models like those we consider, can be defined in several ways. These definitions give the same threshold condition $\left({ℛ}_{0}<1\right)$ for the stability of the disease-free steady-state. Due to the multiphasic nature of vectored disease transmission, differences between definitions of the basic reproduction number for diseases like malaria can often be reconciled by realizing, say, one is the square of the other. A more fundamental issue in defining ${ℛ}_{0}$ for mosquito-borne disease is the complexity that arises from having both human and vectors host the disease agent. Is ${ℛ}_{0}$ the number of secondary mosquito infections due to a small number of primarily infected mosquitoes in an otherwise susceptible population, or the number of secondary human infections due to a small number of initially infected humans, or some combination of the two? While the fates of mosquitoes and humans over the course of an epidemic are coupled, the mosquito- and human-centric basic reproduction numbers are indeed distinct quantities, agreeing only if the disease persists in the population at equilibrium.

To calculate the basic reproduction number ${ℛ}_{0}$ for the pathogen in mosquitoes, we assume that there is a small density of (primary) infected mosquitoes, $M_{i}^{\left(1\right)}(0),M_{i}^{\left(2\right)}(0)$, in patches 1 and 2, respectively, and no infected humans. In this initial phase, the density of susceptible mosquitoes is approximately $M^{\left(1\right)}$ in patch 1 and $M^{\left(2\right)}$ in patch 2, while the numbers of susceptible humans is $H^{\left(1\right)}$ in patch 1 and $H^{\left(2\right)}$ in patch 2. To compute the numbers of secondary infections of mosquitoes in each patch, we must consider two steps: mosquito-to-human followed by human-to-mosquito transmission.

- in patch 1:
  $$H_{i,\text{new}}^{\left(1\right)}=\frac{b_{\text{DD}}H^{\left(1\right)}}{\delta }\cdot \left[c_{11}a_{\text{MH}}^{\left(1\right)}\cdot M_{i}^{\left(1\right)}(0)+c_{12}a_{\text{MH}}^{\left(2\right)}\cdot M_{i}^{\left(2\right)}(0)\right]$$
- in patch 2:
  $$H_{i,\text{new}}^{\left(2\right)}=\frac{b_{\text{DD}}H^{\left(2\right)}}{\delta }\cdot \left[c_{21}a_{\text{MH}}^{\left(1\right)}\cdot M_{i}^{\left(1\right)}(0)+c_{22}a_{\text{MH}}^{\left(2\right)}\cdot M_{i}^{\left(2\right)}(0)\right]$$
- The number of mosquitoes infected by these newly infected humans before they recover is:
  - in patch 1:
    $$M_{i,\text{new}}^{\left(1\right)}=\frac{b_{\text{DD}}a_{\text{HM}}M^{\left(1\right)}}{\gamma }\cdot \left[c_{11}H_{i,\text{new}}^{\left(1\right)}+c_{21}H_{i,\text{new}}^{\left(2\right)}\right]$$
  - in patch 2:
    $$M_{i,\text{new}}^{\left(2\right)}=\frac{b_{\text{DD}}a_{\text{HM}}M^{\left(2\right)}}{\gamma }\cdot \left[c_{12}H_{i,\text{new}}^{\left(1\right)}+c_{22}H_{i,\text{new}}^{\left(2\right)}\right]$$

Note that mosquito death rate *δ* corresponds to mean lifetime 1/δ ; similarly, 1/γ corresponds to the mean time before an infected human recovers. Combining the above two steps allows us to specify patterns of secondary infection (per primary infected mosquito in each patch) in the matrix

$$R=\left[\begin{array}{cc} R(1,1) & R(1,2)\\ R(2,1) & R(2,2) \end{array}\right],$$

where *R*(*j*, *k*) denotes the number of secondary mosquito infections in patch *j* that arose from primarily infected mosquitoes in patch *k*, for j,k∈{1,2}. Consequently, the *j*th row sum gives the number of secondary mosquito infections in patch *j*, and the *k*th column sum is the total number of secondary infections due to initially infected mosquitoes in patch *k*. Tracking the patterns of infection in both patches, we find that

$$\begin{array}{c} R(1,1)=\frac{b_{\text{DD}}^{2}\cdot a_{\text{HM}}\cdot M^{\left(1\right)}\cdot a_{\text{MH}}^{\left(1\right)}}{\gamma \cdot \delta }\cdot [c_{11}^{2}\cdot H^{\left(1\right)}+c_{21}^{2}\cdot H^{\left(2\right)}],\\ R(1,2)=\frac{b_{\text{DD}}^{2}\cdot a_{\text{HM}}\cdot M^{\left(1\right)}\cdot a_{\text{MH}}^{\left(2\right)}}{\gamma \cdot \delta }\cdot [c_{12}\cdot c_{11}\cdot H^{\left(1\right)}+c_{21}\cdot c_{22}\cdot H^{\left(2\right)}],\\ R(2,1)=\frac{b_{\text{DD}}^{2}\cdot a_{\text{HM}}\cdot M^{\left(2\right)}\cdot a_{\text{MH}}^{\left(1\right)}}{\gamma \cdot \delta }\cdot [c_{21}\cdot c_{22}\cdot H^{\left(2\right)}+c_{12}\cdot c_{11}\cdot H^{\left(1\right)}],\\ R(2,2)=\frac{b_{\text{DD}}^{2}\cdot a_{\text{HM}}\cdot M^{\left(2\right)}\cdot a_{\text{MH}}^{\left(2\right)}}{\gamma \cdot \delta }\cdot [c_{22}^{2}\cdot H^{\left(2\right)}+c_{12}^{2}\cdot H^{\left(1\right)}]. \end{array}$$

Note that each of the four secondary transmission terms above has two components: one corresponding to a susceptible human from patch 1 being infected by a primary infected mosquito from the designated patch, and one corresponding to a susceptible human from patch 2 being infected by a primary infected mosquito. Recall that mosquitoes are tied to their patch; only humans visit the other patch. For example, *R*(1,2) records the number of secondary infections of mosquitoes living in patch 1 that arose from a primary infected mosquito in patch 2. There are two patterns of human visitation that can lead to this event. (1) Encoded in the term $c_{12}c_{11}H^{\left(1\right)}$ on the right-hand side of the *R*(1,2) expression: in the first phase, a human in patch 1 visits patch 2 and is infected by a primary mosquito there (and the human returns to its home patch); in the second phase, the newly infected human stays in patch 1 and infects a susceptible mosquito there. (2) Encoded in the term $c_{22}c_{21}H^{\left(2\right)}$ on the right-hand side of the *R*(1,2) expression: in the first phase, a human in patch 2 remains in patch 2 and is infected by a primary mosquito there; in the second phase, the newly infected human visits patch 1 and infects a susceptible mosquito there.

The *basic reproduction number for the density-dependent transmission model* (${ℛ}_{0}^{\text{DD}}$ ) is the leading eigenvalue of the matrix *R*. One can understand this eigenvalue as measuring the number of secondary infections after a ‘generation of infection’—evoking parallels to the theory of discrete-time, age-structured models of population growth, in which the dominant eigenvalue of the Leslie matrix gives the asymptotic growth rate of the population and its associated eigenvector gives the stable age distribution. In a similar manner, the dominant eigenvalue of *R* determines the basic reproduction number of the disease agent globally, and the diagonal entries of *R* give the local reproduction numbers. This ‘risk matrix’ approach is described and used in [11]. The special case of no mosquito-to-human transmission in patch 1 (i.e. $a_{\text{MH}}^{\left(1\right)}=0$) is interesting in that R(1,1)=0=R(2,1) and hence ${ℛ}_{0}^{\text{DD}}=R(2,2)$.

### FD ${ℛ}_{0}$ calculations

Similar to the above case, the calculation of a mosquito-centric ${ℛ}_{0}$ for the FD case begins with an assumption that there is a small density of (primary) infected mosquitoes, $M_{i}^{\left(1\right)}(0),M_{i}^{\left(2\right)}(0)$, in patches 1 and 2, respectively, and no infected humans. In this initial phase, the density of susceptible mosquitoes is approximately $M^{\left(1\right)}$ in patch 1 and $M^{\left(2\right)}$ in patch 2, while the numbers of susceptible humans is $H^{\left(1\right)}$ in patch 1 and $H^{\left(2\right)}$ in patch 2. To compute the numbers of secondary infections of mosquitoes in each patch, we must consider two steps: mosquito-to-human followed by human-to-mosquito transmission.

- in patch 1:
  $$H_{i,\text{new}}^{\left(1\right)}=\frac{b_{\text{FD}}H^{\left(1\right)}}{\delta }\cdot \left[\frac{c_{11}a_{\text{MH}}^{\left(1\right)}}{{\tilde{H}}^{\left(1\right)}}\cdot M_{i}^{\left(1\right)}(0)+\frac{c_{12}a_{\text{MH}}^{\left(2\right)}}{{\tilde{H}}^{\left(2\right)}}\cdot M_{i}^{\left(2\right)}(0)\right]$$
- in patch 2:
  $$H_{i,\text{new}}^{\left(2\right)}=\frac{b_{\text{FD}}H^{\left(2\right)}}{\delta }\cdot \left[\frac{c_{21}a_{\text{MH}}^{\left(1\right)}}{{\tilde{H}}^{\left(1\right)}}\cdot M_{i}^{\left(1\right)}(0)+\frac{c_{22}a_{\text{MH}}^{\left(2\right)}}{{\tilde{H}}^{\left(2\right)}}\cdot M_{i}^{\left(2\right)}(0)\right]$$
- The number of mosquitoes infected by these newly infected humans before they recover is:
  - in patch 1:
    $$M_{i,\text{new}}^{\left(1\right)}=\frac{b_{\text{FD}}a_{\text{HM}}M^{\left(1\right)}}{\gamma }\cdot \frac{c_{11}H_{i,\text{new}}^{\left(1\right)}+c_{21}H_{i,\text{new}}^{\left(2\right)}}{{\tilde{H}}^{\left(1\right)}}$$
  - in patch 2:
    $$M_{i,\text{new}}^{\left(2\right)}=\frac{b_{\text{FD}}a_{\text{HM}}M^{\left(2\right)}}{\gamma }\cdot \frac{c_{12}H_{i,\text{new}}^{\left(1\right)}+c_{22}H_{i,\text{new}}^{\left(2\right)}}{{\tilde{H}}^{\left(2\right)}}$$

Putting these together allows us to specify patterns of secondary infection (per primary infected mosquito in each patch) in the matrix

$$R\prime =\left[\begin{array}{cc} R\prime (1,1) & R\prime (1,2)\\ R\prime (2,1) & R\prime (2,2) \end{array}\right],$$

where *R*′(*j*, *k*) denotes the number of secondary mosquito infections in patch *j* that arose from primary infected mosquitoes in patch *k*, for j,k∈{1,2}. Tracking the patterns of infection in both patches, we arrive at

$$\begin{array}{c} R\prime (1,1)=\frac{b_{\text{FD}}^{2}\cdot a_{\text{HM}}\cdot a_{\text{MH}}^{\left(1\right)}\cdot M^{\left(1\right)}\cdot \left[c_{11}^{2}\cdot H^{\left(1\right)}+c_{21}^{2}\cdot H^{\left(2\right)}\right]}{\delta \cdot \gamma \cdot {\left[{\tilde{H}}^{\left(1\right)}\right]}^{2}},\\ R\prime (1,2)=\frac{b_{\text{FD}}^{2}\cdot a_{\text{HM}}\cdot a_{\text{MH}}^{\left(2\right)}\cdot M^{\left(1\right)}\cdot \left[c_{12}\cdot c_{11}\cdot H^{\left(1\right)}+c_{22}\cdot c_{21}\cdot H^{\left(2\right)}\right]}{\delta \cdot \gamma \cdot {\tilde{H}}^{\left(1\right)}\cdot {\tilde{H}}^{\left(2\right)}},\\ R\prime (2,1)=\frac{b_{\text{FD}}^{2}\cdot a_{\text{HM}}\cdot a_{\text{MH}}^{\left(1\right)}\cdot M^{\left(2\right)}\cdot \left[c_{21}\cdot c_{22}\cdot H^{\left(2\right)}+c_{11}\cdot c_{12}\cdot H^{\left(1\right)}\right]}{\delta \cdot \gamma \cdot {\tilde{H}}^{\left(1\right)}\cdot {\tilde{H}}^{\left(2\right)}},\\ R\prime (2,2)=\frac{b_{\text{FD}}^{2}\cdot a_{\text{HM}}\cdot a_{\text{MH}}^{\left(2\right)}\cdot M^{\left(2\right)}\cdot \left[c_{22}^{2}\cdot H^{\left(2\right)}+c_{12}^{2}\cdot H^{\left(1\right)}\right]}{\delta \cdot \gamma \cdot {\left[{\tilde{H}}^{\left(2\right)}\right]}^{2}}. \end{array}$$

The *basic reproduction number for the frequency-dependent transmission model* (${ℛ}_{0}^{\text{FD}}$) is the leading eigenvalue of the matrix R′. As in the DD transmission model, the special case of no mosquito-to-human transmission in patch 1 (i.e. $a_{\text{MH}}^{\left(1\right)}=0$) results in R′(1,1)=0=R′(2,1) and hence ${ℛ}_{0}^{\text{FD}}=R\prime (2,2)$. If we assume both $a_{\text{MH}}^{\left(1\right)}=0$ and $c_{12}=0$, then we obtain a stark difference between these models: ${ℛ}_{0}^{\text{DD}}=b_{\text{DD}}^{2}a_{\text{MH}}^{\left(2\right)}a_{\text{HM}}M^{\left(1\right)}/\delta \gamma c_{22}^{2}H^{\left(2\right)}$ for the DD model, and ${ℛ}_{0}^{\text{FD}}=b_{\text{FD}}^{2}a_{\text{MH}}^{\left(2\right)}a_{\text{HM}}M^{\left(2\right)}/\delta \gamma H^{\left(2\right)}$ for the FD model. Thus, one-way visitation to a patch with complete suppression of transmission from mosquitoes to humans has a strong effect in the DD model, but no effect in the FD model. In fact, the latter ${ℛ}_{0}^{\text{FD}}$ is in the standard form for a Ross–Macdonald model with no patch structure.

Notice that mosquito and human densities in the terms characterizing the basic reproduction number in the FD model appear in ratio form M/H, while in the DD model they appear in product form MH.

Our ${ℛ}_{0}$ calculations, for both DD and FD transmission, were based on computing numbers of secondarily infected mosquitoes that arose from the primary mosquito infections. Since human and mosquito infections are intertwined due to the nature of vector transmission, it should not be surprising that the threshold ${ℛ}_{0}=1$ above which human infection persists is the same as the one that guarantees persistence of mosquito infection. In numerical solutions of our differential equations (not shown), we saw positive equilibrium densities of both infected mosquitoes and infected humans precisely when ${ℛ}_{0}>1$.

## Supplementary Material

eoaa035_Supplementary_Data
